# Medical Artificial Intelligence for Rural Areas of Developing Countries a Boon or Bane: A Contemporary Narrative Review

**DOI:** 10.1002/hsr2.71518

**Published:** 2026-11-10

**Authors:** Shraddha Gurha, Suyesh Shrivastava, Prashant Suravajhala, Pushpendra Singh

**Affiliations:** ^1^ Model Rural Health Research Unit (MRHRU) Badoni Madhya Pradesh India; ^2^ ICMR‐National Institute of Research in Tribal Health (NIRTH) Jabalpur Madhya Pradesh India; ^3^ Department of Biosciences Manipal University Jaipur Dehmi Kalan Rajasthan India; ^4^ Bioclues.org Hyderabad India

**Keywords:** artificial intelligence (AI), ethical considerations, healthcare access, healthcare challenges, healthcare inequities, implementation, rural healthcare, rural medical education

## Abstract

**Background and Aim:**

The integration of medical artificial intelligence (AI) in rural India holds transformative potential for enhancing healthcare access and quality. This narrative review examines the promising applications of medical AI in addressing healthcare disparities, increasing service efficiency, and mitigating physician shortages in underserved areas. However, challenges persist, including medical students' limited awareness and education about AI, reluctance to engage in rural health services, and migration trends affecting healthcare delivery.

**Methodology:**

Effective implementation demands a multifaceted approach, tackling technological, economic, and contextual obstacles through stakeholder collaboration. Ethical considerations such as bias, data privacy, and local workforce involvement are paramount for the responsible deployment of medical AI.

**Results and Conclusion:**

This review delves into both the opportunities and challenges, emphasizing the importance of addressing these issues through enhanced education and ethical frameworks. Ultimately, a balanced strategy is essential to harness the potential of medical AI in revolutionizing rural healthcare, highlighting the need for innovative policies that align technological advancements with community needs.

## 1 Introduction

Medical artificial intelligence (AI) can enhance healthcare delivery, especially in resource‐limited nations where high‐quality care is scarce. AI has been shown to improve clinical standards, efficiency, reduce medical errors, and enhance treatment quality, particularly in remote areas of developing countries [1]. In densely populated countries with limited healthcare resources, AI‐assisted medicine can significantly improve healthcare outcomes and address workforce upskilling needs [2, 3]. AI can revolutionize healthcare by enabling innovative and efficient care delivery, crucial in developing countries with limited medical resources [4]. Despite its potential, barriers such as ethical concerns, transparency, and algorithmic bias hinder AI's use in healthcare [5]. Nevertheless, new AI models are increasingly integrated into healthcare processes, highlighting the gap between AI's potential and its practical application [6]. AI in healthcare can address accessibility, appeal, and availability issues in underdeveloped nations [7]. AI and machine learning can improve cardiovascular health in low‐ and middle‐income countries [8]. AI's diverse applications will significantly enhance equitable healthcare delivery in developing nations, particularly in addressing access to healthcare professionals and services [9]. While AI's potential to transform healthcare in developing countries is clear, further research, ethical considerations, and practical implementation strategies are necessary to fully realize its benefits. This narrative review explores medical AI deployment in rural India, examining the opportunities, implementation challenges, and ethical considerations in culturally diverse and resource‐limited settings. It provides a comprehensive assessment of whether medical AI can benefit rural healthcare in India. It contributes to the ongoing discussion on the intersection of technology and healthcare in developing countries, focusing on the unique issues faced by India's rural population.

## 2 Methodology and Analysis of AI in Healthcare

AI is becoming essential in healthcare, particularly in intelligent clinical decision support systems (CDSS) that analyze vast patient data using AI algorithms and machine learning [10]. Research demonstrates the effectiveness of deep learning algorithms in detecting skin cancer [11] and diabetic retinopathy [12]. Computer‐aided diagnostics improve assessments across specialties, with models like DeepFocus [13] and HistoQC [14] assisting pathologists in identifying poorly defined areas in digital slides. However, challenges such as data interoperability, limited integration with electronic health records (EHRs), and liability issues hinder the growth of these systems. AI also has potential in drug discovery, but faces data availability obstacles and regulatory complexities requiring cross‐disciplinary collaboration. Machine learning advances cardiovascular disease (CVD) evaluations, with models analyzing large data sets showing promise in predicting CVD risks [15]. Precision medicine aims to tailor interventions based on individual factors, with AI enhancing pathologists' ability to detect disease markers earlier [9]. AI‐driven predictive analytics offer vast potential for precision medicine, but everyday clinical application remains limited. Studies have evaluated breast cancer prediction algorithms trained on mammography images and comprehensive health data, achieving accuracy comparable to expert radiologists [16]. AI could also reduce interval cancers in mammography, identifying cases in earlier scans with minimal malignancy signs [17]. As genomic and clinical data sets grow alongside AI algorithm advancements, prospects for precision medicine are increasingly attainable.

## 3 Opportunities for Medical AI in Rural India

In developing countries such as India, the use of medical AI has huge potential for enhancing healthcare access and service quality [18]. By teaching other healthcare professionals to apply AI techniques, medical AI technology can enhance the quality of medical services, increase physician output, and reduce healthcare disparities. This would help compensate for the dearth of physicians in rural areas [18]. According to doctors' opinions and contentment with AI in cancer therapy, especially in low‐ and middle‐income nations such as India, AI technologies have the potential to supply much‐needed specialized treatment expertise to satisfy population demands, especially in rural locations [19].

Initiatives such as developing a distinct medical graduate course for students from rural backgrounds in India have been recommended to cater to the needs of rural people to alleviate the shortfall in the health system [20]. However, there are issues with AI awareness and education among medical students in India, emphasizing the significance of measuring and improving AI understanding in medical education [21, 22]. Furthermore, medical students' reluctance to join rural healthcare in India is influenced by variables such as insufficient facilities, accommodation, and salary, highlighting the need to overcome these barriers to lure more students to rural services [23]. The shortage of health personnel, particularly doctors, has long affected India's rural health system and has highlighted the importance of understanding and addressing barriers to working in rural areas [24]. Furthermore, changes in rural India's consumption, production, and income patterns present considerable prospects, showing the possibility for rural marketing innovations to tap into the enormous rural market and its opportunities [25]. Comprehending India's low rate of rural‐to‐urban migration is crucial since it could be impacted by elements such as the closing gap between rural and urban health, infrastructure, employment opportunities, and economic conditions [26]. In summary, opportunities for medical AI in rural India are vast, with the potential to enhance access to healthcare, address healthcare inequities, and improve the quality of medical services (Figure 1). Table 1 illustrates various opportunities for medical AI in rural India.

**Figure 1:**
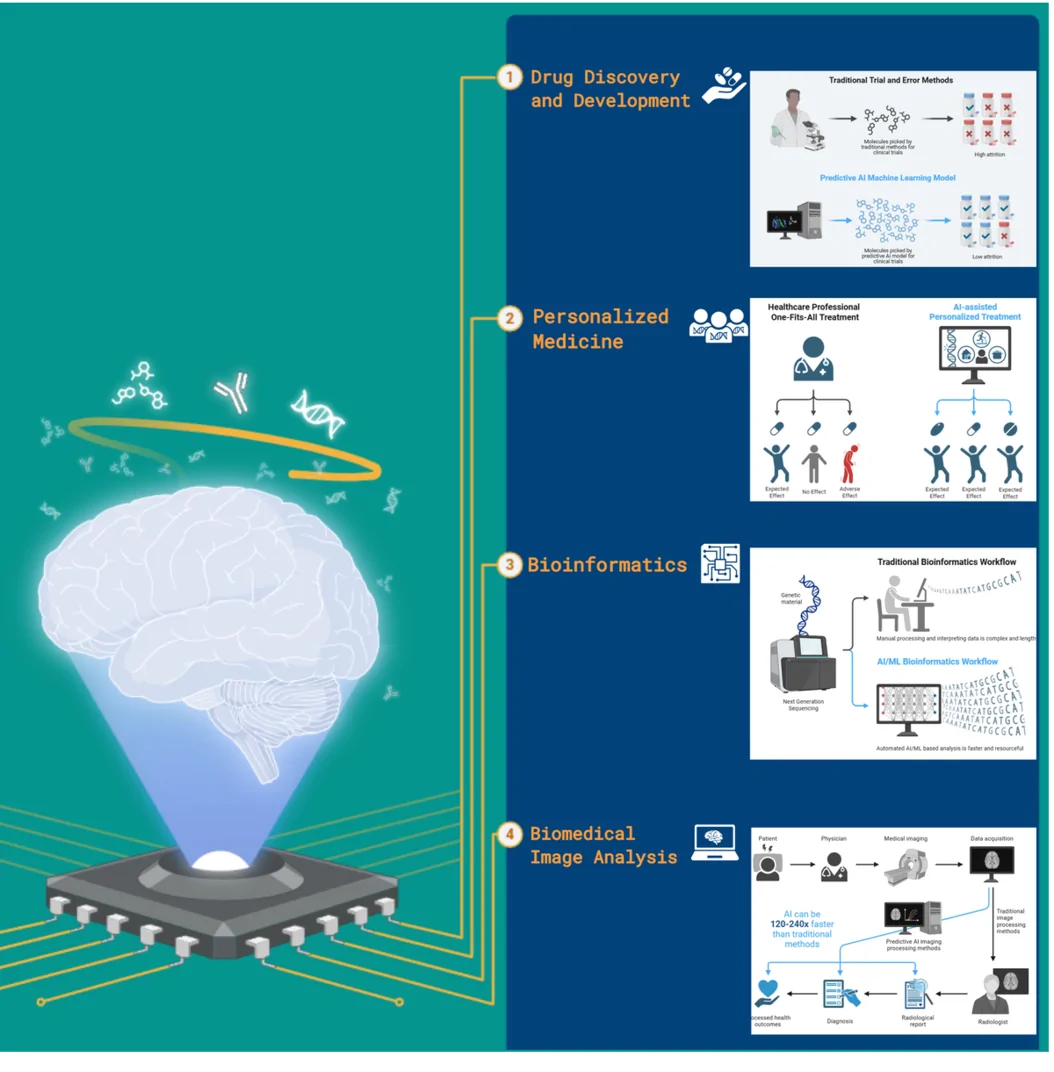
Medical artificial intelligence: opportunities to implementation (created in https://BioRender.com).

**Table 1:** Rural health revolution: AI opportunities unveiled.

No.	Opportunity	Description
1	Health tech breakthrough	Improving rural India's healthcare access and service quality through AI‐driven efficiency and workforce training [10].
2	Cancer care boost	Empowering rural healthcare with AI tools providing vital subspecialty treatment information, especially in cancer treatment [11].
3	Medical education mavericks	Novel concepts for targeted medical graduate courses aimed at bridging rural health system gaps [12].
4	Knowledge kinks	Unpacking barriers in AI knowledge and teaching among Indian medical students, requiring assessment and improvement initiatives [13, 14].
5	Rural service rally	Addressing medical students' unwillingness with comprehensive solutions to rural service barriers [15].

## 4 Challenges in Implementing Medical AI in Rural India

The implementation of medical AI in rural India involves multiple challenges that must be handled carefully. AI has attracted considerable attention in the healthcare industry, and its implementation issues have not been sufficiently addressed [27]. One of the limitations of implementing AI in medical education is the difficulty in determining its effectiveness and the technological issues in building AI applications [22]. Moreover, challenges such as data privacy and sharing, algorithm transparency, data standardization, and platform interoperability, in addition to patient safety issues, impede the practical application of AI in medicine [28]. AI start‐ups in India provide healthcare solutions [29], but the digital divide between rural and urban areas hinders AI implementation in healthcare [30]. To realize substantial benefits for the healthcare sector, it is imperative for the government to address the challenges associated with the implementation of AI in India [31]. A Four‐AI‐Challenges Model has been proposed to incorporate major dimensions and sub‐aspects of the significant challenges associated with AI implementation in the public sector. This model offers public managers tasked with implementing AI as well as tangible implications for research related to AI [32]. The challenges in implementing medical AI in rural India are presented in Table 2. Intervention of indigenous populations is pivotal in addressing the challenges associated with implementing AI in rural communities. By engaging indigenous populations in the development and deployment of AI technologies, it is possible to integrate local knowledge and cultural perspectives, thereby ensuring that solutions are contextually appropriate and sustainable [33]. This approach aids in overcoming obstacles such as limited digital infrastructure, low technological literacy, and potential resistance to new technologies [34]. Indigenous interventions also facilitate the identification of specific community needs and priorities, enabling the creation of tailored AI applications that directly benefit rural inhabitants [35]. Moreover, it promotes capacity building and empowerment within these communities, fostering the long‐term adoption and maintenance of AI systems [35]. Nonetheless, this process necessitates careful consideration of ethical, legal, and social issues/implications, data sovereignty, and the preservation of traditional practices alongside technological advancements.

**Table 2:** Challenges of medical AI in rural healthcare.

No.	Challenge	Description
1	Tech turbulence	Addressing the hurdles of AI implementation in healthcare, from assessing effectiveness to data sharing challenges [19, 20].
2	AI‐CDSS dilemmas	Unraveling tensions in deploying AI‐CDSS in rural clinics, balancing local context, usability, and transparency [21].
3	Digital divide dilemma	Bridging the technological gap between urban and rural areas is a barrier in efficiently using and deploying AI in healthcare [24].
4	Finance frontiers	Confronting financial challenges in promoting medical AI technology in rural areas of developing countries [1].

## 5 Ethical Issues in Implementing Medical AI in Rural India

In rural India, the application of medical AI raises unique ethical issues that need to be properly managed to protect the community's health. In rural parts of developing nations, medical AI has the potential to greatly enhance healthcare access and service quality [1]. But it is important to address concerns about bias, data privacy, and how AI may affect employment opportunities [36]. In the process of developing ethical rules for AI application, it is imperative to give local communities' needs and values top priority and to collaborate with local stakeholders [36]. One practical method for incorporating substantial ethical considerations into the medical AI design process is the “embedded ethics” concept [37]. Public perspectives of the use of AI in healthcare emphasize the significance of examining ethical problems throughout medical AI research to achieve optimum benefit to human well‐being [38]. Furthermore, to address the ethical problems connected with AI adoption in healthcare, the establishment and application of ethical principles for AI is advocated [28]. Achieving a data‐driven risk assessment technique for ethical AI is critical, as it necessitates legal, ethical, and societal considerations to sustain human values in a data‐driven era [39]. Geographic disparities in the distribution of the health workforce offer substantial hurdles to the deployment of medical AI in rural India [40]. Due to a physician shortage in rural regions, additional health workers must be trained to apply AI medical practices to compensate, underscoring the necessity of ethical considerations in assuring the right use of AI in healthcare delivery [1]. Furthermore, the constraints connected with the adoption of AI‐powered CDSS in rural clinics highlight the need for ethical AI solutions that are transparent and trustworthy, as well as align with the local context and workflow [29]. The ethical issues surrounding the implementation of medical AI in rural India go beyond the technology itself. Medical students' and young doctors' attitudes toward rural service are critical factors of the success of AI adoption in rural healthcare [41]. Understanding the factors that influence the decision to work in rural health services and addressing the barriers that impede working in rural regions are critical for effective human resource planning in rural healthcare [42, 43, 44]. Furthermore, the ethical implications of teleophthalmology as a model for enhancing eye care delivery in India's rural and disadvantaged areas underline the importance of ensuring fair access to AI‐enabled healthcare services [45]. In summary, the ethical considerations in adopting medical AI in rural India are diverse and necessitate a multidisciplinary approach that addresses not only the technology components but also the sociological, cultural, and workforce‐related challenges. Engaging with local populations, including ethics into AI development, and comprehending the attitudes of healthcare professionals and students are all necessary for the ethical and effective application of medical AI in rural India. Table 3 presents the ethical considerations in implementing medical AI in rural India.

**Table 3:** AI with integrity: ethical considerations in rural health tech.

No.	Ethical consideration	Description
1	Bias, Privacy, and Labor	Uncovering issues of ethics such as prejudice and data privacy, as well as the labor market impact of AI implementation in rural India [28].
2	“Embedded Ethics” Odyssey	Charting a course for ethical AI through an “embedded ethics” approach, integrating ethics from the start of development [29].
3	Workforce Woes	Ethical challenges in addressing geographical health workforce imbalances and ensuring fair AI‐enabled healthcare access [32].
4	Telehealth Terrain	Managing the moral ramifications of teleophthalmology and guaranteeing fair access to AI‐powered medical services [37].

## 6 Future Directions Medical AI in Rural India

Integrating medical AI into rural India presents both opportunities and challenges. AI can improve healthcare quality and efficiency [46]. Medical students struggle to practice in rural areas because of insufficient resources and infrastructure [32]. Assessing AI knowledge and education among physicians and Indian medical students is crucial for rural healthcare professionals [47]. AI can accelerate case identification, screening, contact tracing, and diagnosis during health crises such as COVID‐19, particularly in resource‐limited rural areas [48]. The cost‐effectiveness of AI‐based diabetic retinopathy screening highlights AI's potential in rural healthcare [49]. However, challenges in integrating AI into rural healthcare include forming clinical partnerships for chronic condition management via mHealth platforms [50]. Enhancing AI education in medical curricula is vital to address medical students' perceptions of AI and radiology [44]. Advancing medical AI in rural India requires the following: customizing AI for rural health, considering local issues, prevalent diseases, and infrastructure limitations. Developing programs to attract medical professionals to rural areas and incorporating AI to improve healthcare quality amid resource constraints [42, 51]. Provide specialized training for rural healthcare providers to use AI effectively for better decision‐making and patient care. Leveraging AI to address health crises like COVID‐19 and managing issues such as diabetic retinopathy through early detection and AI‐driven solutions [41, 51]. NITI Aayog's Contribution to AI in Healthcare Advancement in India Recognizing AI's transformative impact, NITI Aayog released a discussion paper in June 2018 on the National Strategy for Artificial Intelligence (NSAI), emphasizing AI's potential for economic and societal benefits under “AI for All.” The NSAI suggests developing India's AI ecosystem by promoting research, reskilling the workforce, encouraging AI solutions, and establishing guidelines for “responsible AI,” highlighting private sector roles and government intervention areas [52]. In the 2018–2019 budget, India's Finance Minister directed NITI Aayog to create the National Programme on AI, which aims to advance AI research and applications, addressing India's unique challenges across sectors by fostering collaboration among academic institutions, industries, and government bodies [53]. This initiative seeks to position India at the forefront of AI by promoting innovation, economic growth, and societal solutions. NITI Aayog's AI Initiatives in Healthcare: NITI Aayog addresses India's complex healthcare system with AI‐driven solutions by collaborating with a major AI technology company on critical projects. The global AI healthcare sector is projected to grow by 40% annually, reaching USD 6.6 billion by 2021. Key Initiatives include, Oncology Screening: creating a national database of annotated pathological images to enhance early cancer detection and treatment, Diabetic Eye Disease partnering with Microsoft and Forus Health to trial early diabetic retinopathy detection using AI, as portable “3Nethra” eye examination device for underserved areas [46]. Table 4 presents Beyond Tomorrow: Charting the Future of Rural Health AI.

**Table 4:** Beyond Tomorrow: Charting the Future of Rural Health AI.

No.	Direction	Description
1	Rural renaissance	Overcoming barriers that deter medical students from serving in rural areas, as well as filling resource and infrastructure shortfalls [39].
2	AI perspectives palette	Unifying doctors' and medical students' AI knowledge and views for a more harmonious healthcare future [40].
3	AI in crisis control	Harnessing AI's potential in combating health crises, speeding up identification, screening, and diagnosis in rural India [41].
4	Cost‐effective care	Demonstrating the cost‐effectiveness of AI‐based screening for specific healthcare needs in rural areas [42].

## 7 E‐Health Technologies and Digital Transformation in Indian Healthcare

India's healthcare system is undergoing a digital transformation to enhance services and accessibility through e‐health technologies. Government initiatives like NITI Aayog's National Health Stack and the Ministry of Health and Family Welfare's National Digital Health Blueprint are key drivers. A central component is a single health identifier, which consolidates medical records, improves data management, accelerates diagnostics, and enables comprehensive patient care, promoting geographic flexibility and ensuring access to quality healthcare regardless of location [54, 55]. Remote digital consultations are crucial, improving access to medical professionals and easing the burden on physical facilities, especially in regions with geographical barriers. Digital advancements standardize healthcare delivery through uniform protocols, ensuring consistent, high‐quality care, and reducing disparities [56]. This shift enhances efficiency and reduces costs by streamlining administrative tasks, benefiting both providers and patients [57]. Personal EHRs facilitate informed decisions about well‐being and extend healthcare beyond traditional boundaries by focusing on preventive measures and overall wellness, transforming care from reactive to holistic maintenance [58].

## 8 Feasibility Studies and Pilot Interventions

NITI Aayog has explored incorporating AI in public healthcare, sponsoring studies to evaluate AI applications, including Elsevier's “Arezzo” with Indian Standard Treatment Guidelines. The research showed the potential to convert guidelines into an AI‐powered decision support system, assessing content accuracy, usability, and adherence to medical guidelines. Utilizing “Arezzo” demonstrated AI's capability to revolutionize healthcare procedures, adhering to accepted practices and providing insights into advanced technologies' practicality in real‐world settings. These findings underscore AI's potential to enhance healthcare practices, highlighting future AI integration in healthcare decision‐making processes [59].

## 9 Blockchain Technologies in the Realm of Democratizing Big Data

Blockchain and AI are essential for democratizing big data. As data dissemination grows, the decentralization of large networks requires blockchain and AI to capture small information pieces securely. These technologies can manage public health data and EHRs without compromising ethics or privacy. During the COVID‐19 pandemic, standardizing EHR data involved blockchain and AI to monitor outbreaks, similar to approaches used in Zika, Ebola, and other epidemics. Healthcare providers employed haptics, cryptography, and contact tracing/transmission pattern surveillance. Since COVID‐19's onset, numerous attempts have utilized these technologies with Long Short‐Term Memory and dynamic studies, improving digital health and well‐being through democratized healthcare [56].

## 10 AI in Healthcare: Obstacles and Limitations

AI promises improved patient care but faces barriers in healthcare integration. A primary concern is data protection. Healthcare institutions hold sensitive patient information, making privacy and security critical for governments, providers, and patients [26]. Ensuring robust data governance, proper data collection, and explicit consent is essential to build patient trust and leverage data for AI progress [27]. Stringent healthcare regulations ensure safety but can delay AI innovation adoption [28]. The influx of new AI technologies raises questions about their necessity and efficacy in current treatments. Data breaches can severely erode trust, lead to legal issues, and harm patient health [29]. The potential of AI in healthcare is substantial, but its widespread adoption faces several challenges. A primary issue is the need for extensive, high‐quality data. AI algorithms require large, varied data sets for training and validation, yet obtaining these is difficult due to privacy concerns and regulatory constraints, leading to isolated data pools that limit AI's effectiveness. Additionally, biases in healthcare data can result in unfair and inaccurate AI predictions, especially when certain groups are underrepresented. Another constraint is the risk of excessive dependence and automation bias. While AI offers valuable insights and predictions, it should not replace human expertise. Overreliance on AI might cause healthcare providers to become complacent, potentially overlooking crucial clinical information or making errors based solely on AI suggestions. Balancing AI capabilities with human judgment is a complex challenge. Moreover, AI algorithms often lack transparency, making it difficult for healthcare professionals to understand AI‐driven recommendations, which can erode trust in the technology. Addressing these issues requires collaboration among healthcare experts, data scientists, and policymakers to ensure AI's safe and effective use. Ethical and legal limitations also impede AI's progress in healthcare [57]. Questions of liability and accountability arise as AI systems become more integrated into clinical practice. It is unclear whether responsibility for medical errors or incorrect diagnoses should fall on the healthcare provider, the algorithm developers, or the implementing organization. Establishing clear legal frameworks and ethical guidelines is essential to ensure patient safety and mitigate potential legal disputes. The cost of implementing AI in healthcare presents another significant limitation [58]. Developing and maintaining AI solutions and training healthcare professionals to use them effectively is expensive. Smaller healthcare institutions and resource‐constrained regions may struggle to afford these technologies, leading to disparities in access to AI‐driven healthcare benefits. Additionally, the rapid pace of AI advancements requires continuous investment in updates and training, further increasing financial burdens. Balancing the benefits of AI against associated costs is crucial for healthcare decision‐makers [59] (created in https://BioRender.com).

## 11 Conclusions

In rural India, the integration of medical AI brings both opportunities and challenges. Medical AI has the ability to improve rural healthcare access, address inequities, and improve service quality. However, its adoption necessitates consideration of technical, economic, and ethical issues. The role of NITI Aayog in steering this transformation is critical, as evidenced by its National Programme on AI and initiatives in AI healthcare solutions. Bridging knowledge gaps, focusing on cost‐effective solutions, and advocating for thorough training are essential for unleashing the full promise of medical AI in rural India. A balanced strategy integrating technological innovation, ethical considerations, and proactive policies is required to effectively reshape rural healthcare.

## Author Contributions

**Shraddha Gurha:** conceptualization, methodology, data curation, writing – original draft, writing – review and editing. **Suyesh Shrivastava:** validation, methodology, supervision, writing – review and editing, visualization. **Prashanth Suravajhala:** conceptualization, methodology, data curation, visualization, validation, supervision, writing – review and editing. **Pushpendra Singh:** conceptualization, methodology, data curation, validation, visualization, supervision, resources, writing – review and editing.

## Disclosure

The lead author Dr Shraddha Gurha, and Dr Suyesh Shrivastava, Prof Prashant Suravajhala and Dr Pushpendra Singh affirm that this manuscript is an honest, accurate, and transparent account of the study being reported; that no important aspects of the study have been omitted; and that any discrepancies from the study as planned (and, if relevant, registered) have been explained.

## Ethics Statement

The authors have nothing to report.

## Consent

The authors have nothing to report.

## Conflicts of Interest

The authors declare no conflicts of interest.

## Data Availability

The data utilized in this narrative review are not publicly available due to privacy and ethical considerations concerning sensitive health information. Researchers interested in accessing the data set should contact the corresponding author with a formal request detailing their proposed use.
